# Supplementary figures and images for: Identification and characterization of auxin response factor (ARF) family members involved in fig (Ficus carica L.) fruit development
Source: PeerJ. 2022 Jul 22;10:e13798. doi: 10.7717/peerj.13798 (PMC9310797; doi:10.7717/peerj.13798)

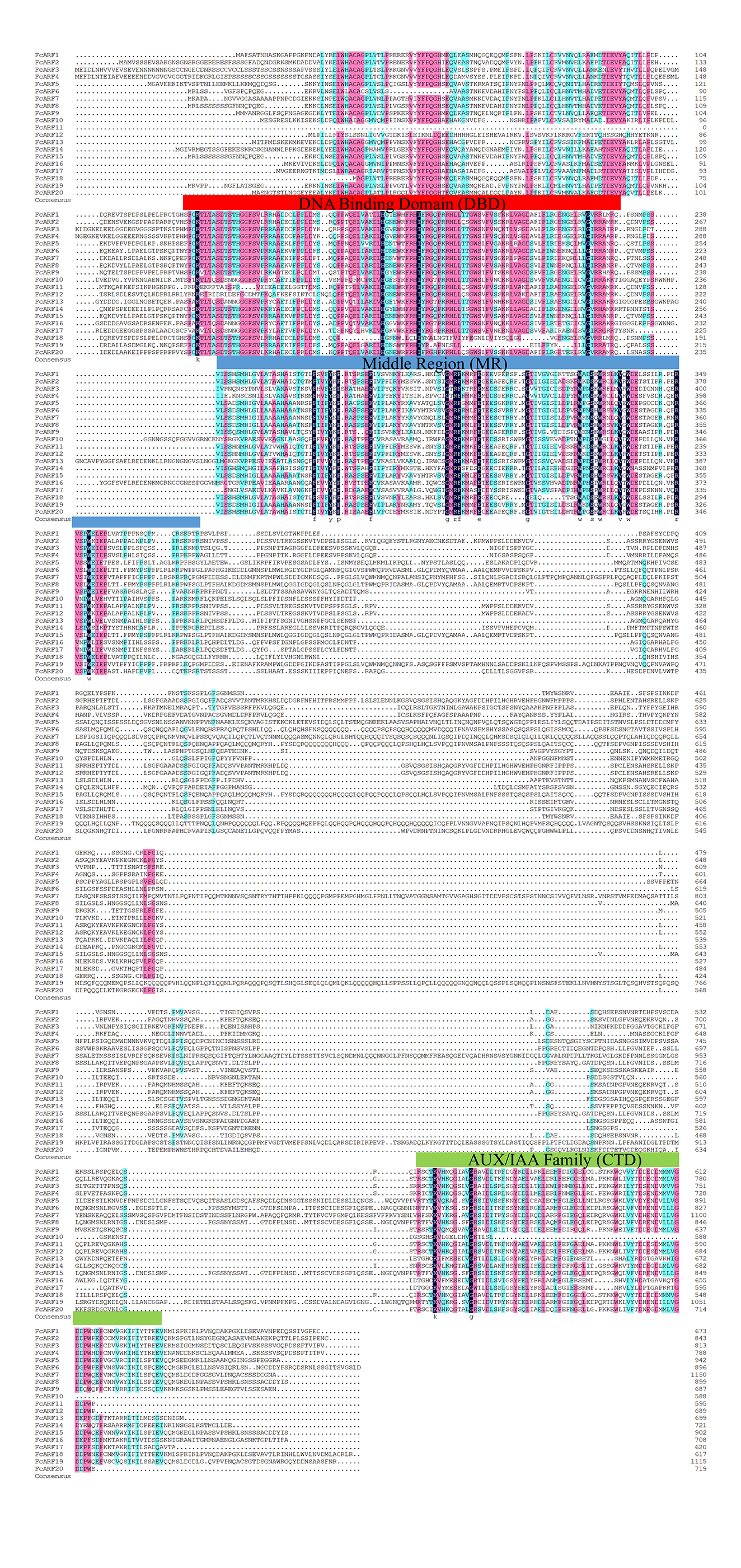

Supplement: Supplemental Information 1 [file peerj-10-13798-s001.png]

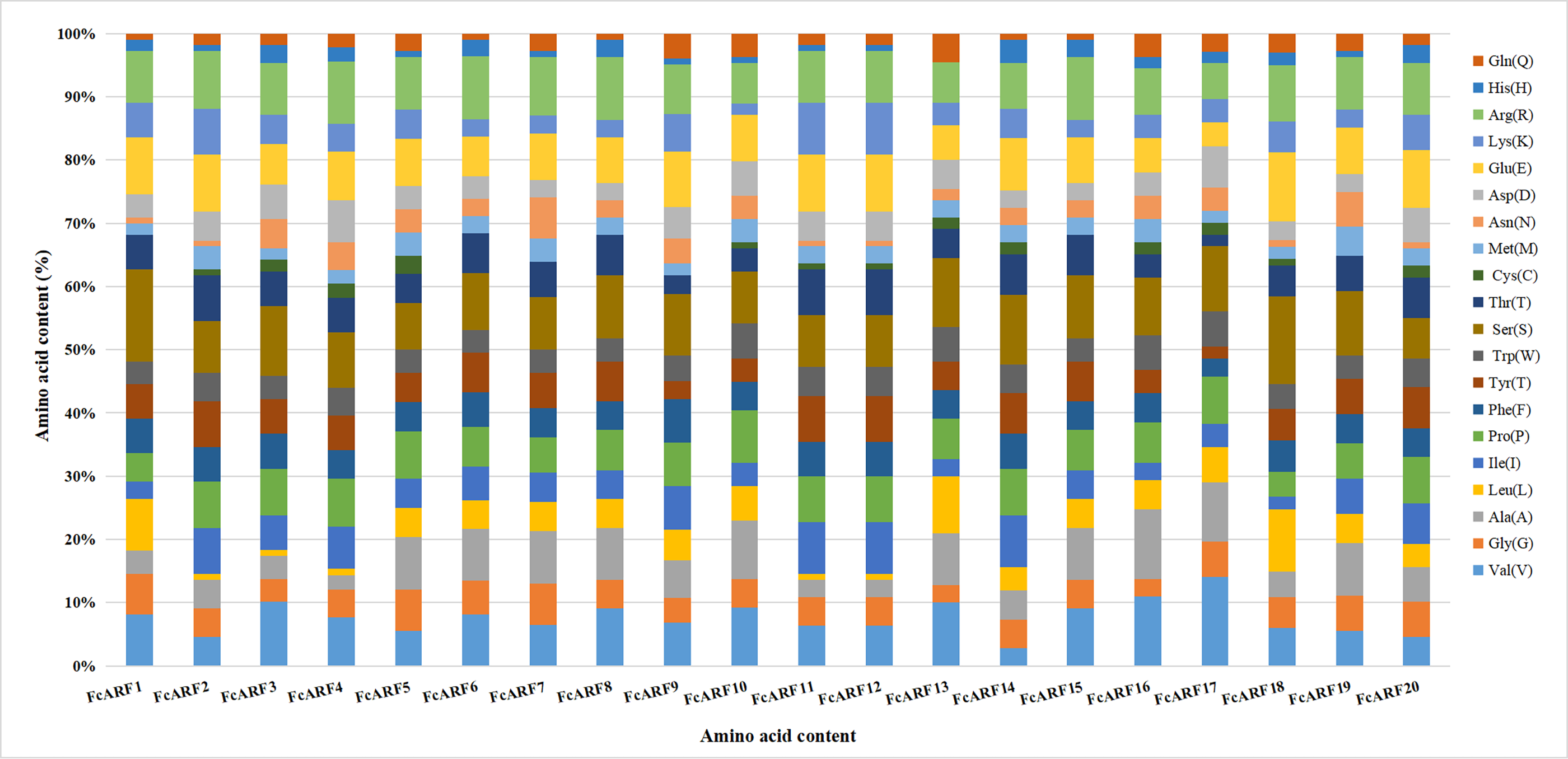

Supplement: Supplemental Information 2 [file peerj-10-13798-s002.png]

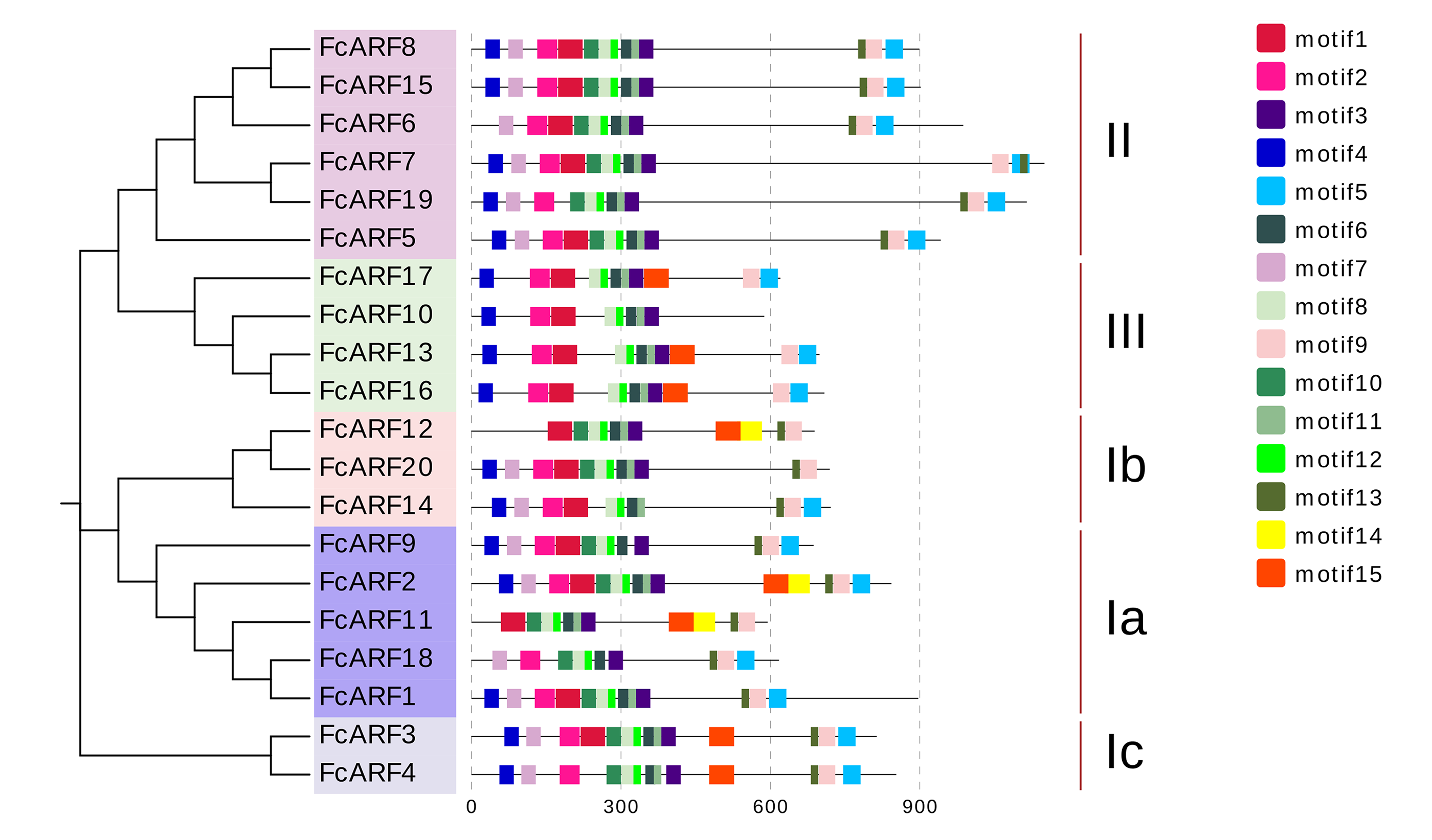

Supplement: Supplemental Information 3 [file peerj-10-13798-s003.png]

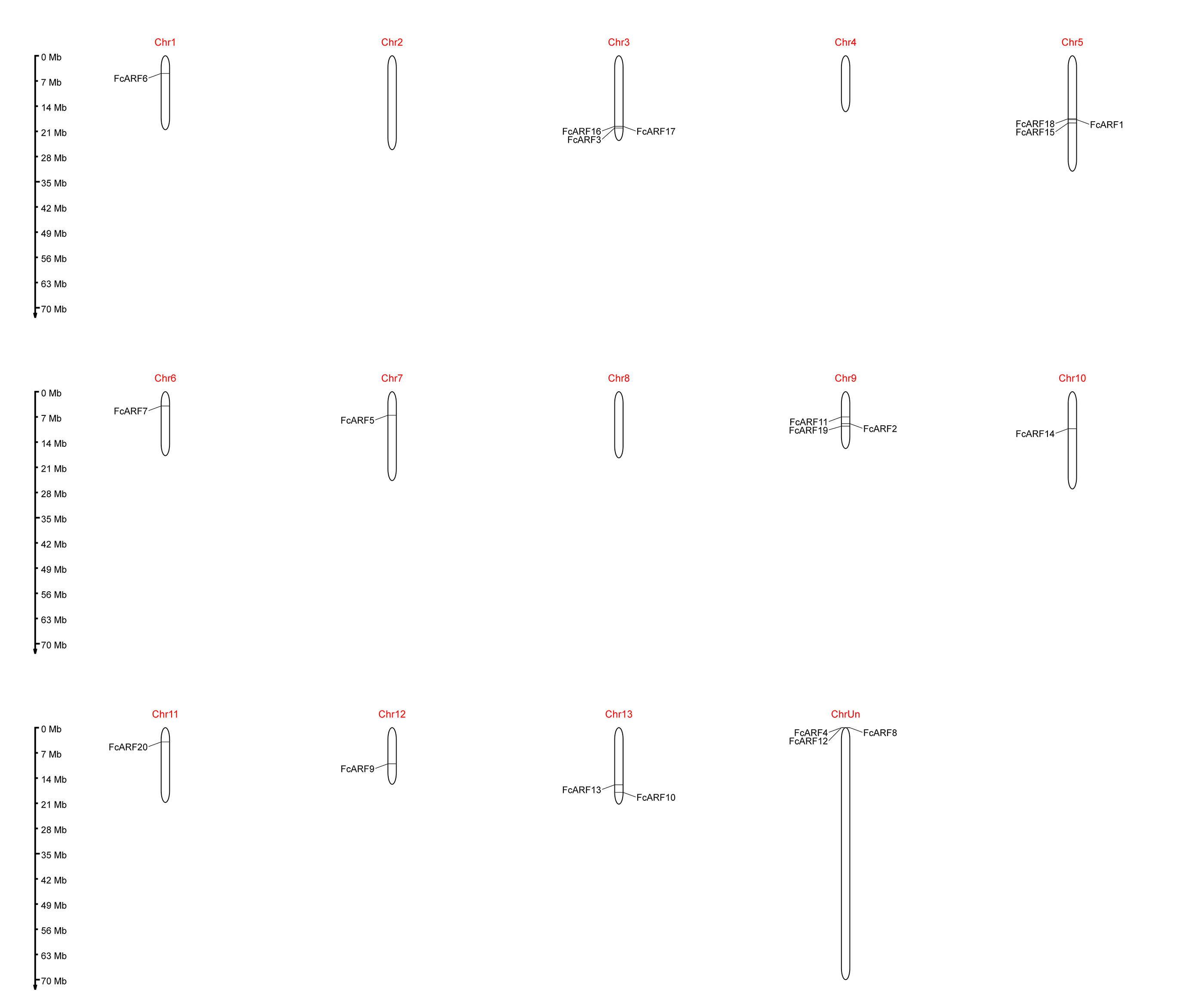

Supplement: Supplemental Information 4 [file peerj-10-13798-s004.png]

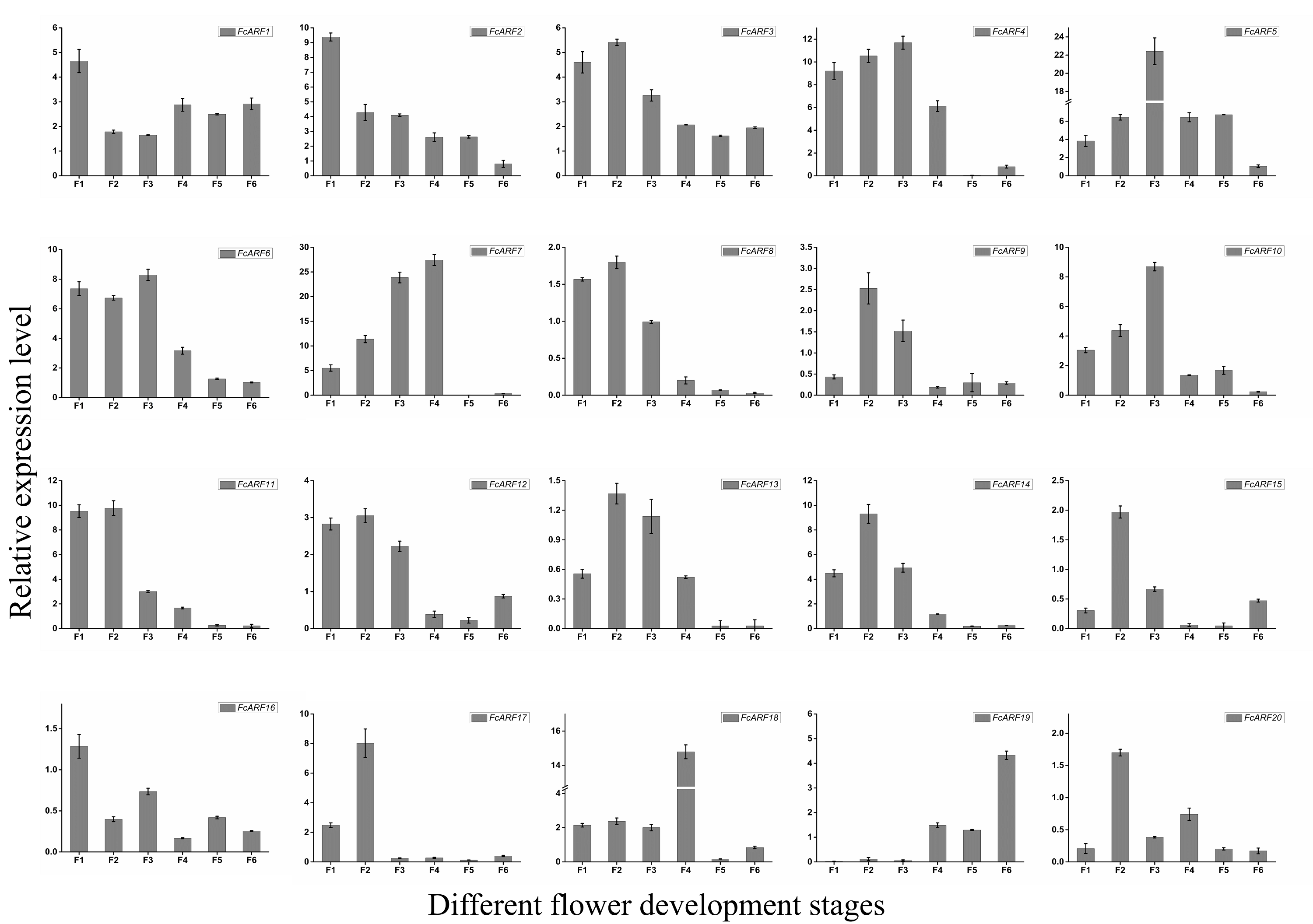

Supplement: Supplemental Information 5 [file peerj-10-13798-s005.png]

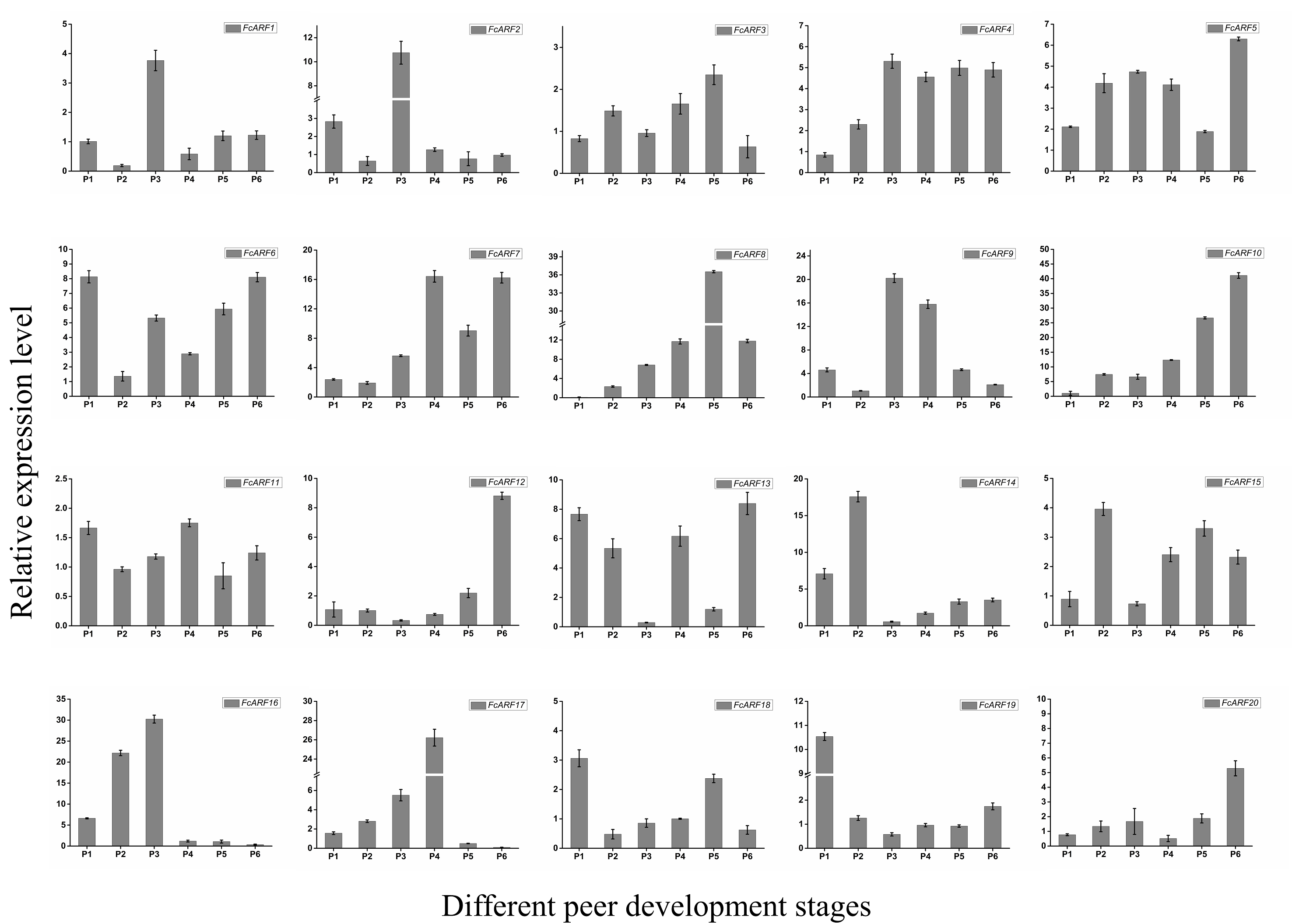

Supplement: Supplemental Information 6 [file peerj-10-13798-s006.png]
