# Supplementary material for: Identification and characterization of auxin response factor (ARF) family members involved in fig (Ficus carica L.) fruit development
Source: PeerJ. 2022 Jul 22;10:e13798. doi: 10.7717/peerj.13798 (PMC9310797; doi:10.7717/peerj.13798)
Supplement: Supplemental Information 7 [file peerj-10-13798-s007.docx]

**Supplementary Table S1 Spatiotemporal expression of ARF gene family in common plants and their roles in growth and development**

| **Tissue** | **Species** | **Gene name** | **Fuction** | **Proven target genes** | **References** |
| --- | --- | --- | --- | --- | --- |
| **Root** | ***Arabidopsis thaliana*** | *AtARF10/16* | auxin related phenotype, no lateral roots | GSR1 | Wang et al., 2005; Y16  et al., 2016 |
|  |  | *AtARF11* | promoting lateral root formation in hormone pathways |  | Okushima et al., 2005; Zhang et al., 2020 |
|  |  | *AtARF7/19* | act redundantly with in controlling lateral root growth | PRH1 |  |
|  | ***Oryza sativa*** | *OsARF5* | root cap growth and development and lateral root | IAA19 | Wang et al., 2010; Luo et al., 2015; |
|  |  | *OsARF6/17* |  | ILA1 |  |
|  |  | *OsARF12/1625* |  |  |  |
|  | ***Citrus sinensis*** | *CiARF7/9/16/19* | high expression in roots |  | Li et al., 2016 |
| **Steem** | ***Nicotiana tabacum L.*** | *NtARF8/17/19* | Regulate stem growth | TTG2 | Ge et al., 2016 |
|  |  | *NtARF1-13/15-19/21-30/32-39/42-46* | highly expressed in stem tissue |  | Zhang et al., 2021 |
|  | ***Citrus sinensis*** | *CiARF1-6/10/15/17/18* | highly expressed in stem tissue |  | Li et al., 2016 |
| **Leaves** | ***Arabidopsis thaliana*** | *AtARF1/2* | control leaf senescence | ORE14 | Ellis et al. 2005; Lim et al., 2010 |
|  |  | *AtARF3* | leaf polarity specification | KANADI | Kelley et al., 2012 |
|  |  | *AtARF5* | critically required for embryonic root | DAD1 | Krogan et al. 2014; Zhang et al., 2020 |
|  |  | *AtARF7/19* | controlling leaf expansion | PRH1 |  |
|  | ***Nicotiana tabacum L.*** | *NtARF1/2/4-19/21-33/35-39/43-46* | highly expressed in old leaves |  | Zhang et al., 2021 |
|  | ***Oryza sativa*** | *OsARF19* | control leaf Angle of rice | BRI1 | Shen et al., 2010 |
| **Flower** | ***Arabidopsis thaliana*** | *AtARF1* | control leaf senescence and floral organ abscission |  | Ellis et al. 2005 |
|  |  | *AtARF2* | floral organs developing  light-grown and dark-grown seedlings | WUS/AG/AP2 | Okushima et al., 2005 |
|  |  | *AtARF4* | role in organ polarity |  | Pekker et al. 2005； |
|  |  | *AtARF5* | flower formation | DAD1 | Krogan et al.，2014 |
|  |  | *AtARF17* | regulation pollen wall structure | CalS5 | Yang et al., 2013 |
|  |  | *AtARF6* | flower maturation | DAD1 | Goetz et al.，2006 |
|  |  | *AtARF8* | flower maturation; regulate fertilization |  |  |
|  |  | *AtARF12* | seeds developing |  | Okushima et al., 2005 |
|  | ***Prunus mume*** | *PmARF1/2/5-7/13/17* | high expression in complete flower bud |  | Song et al., 2015 |
|  |  | *PmARF11* | high expression in pistil than stamen |  |  |
|  | ***Citrus sinensis*** | *CiARF3/5-7/9/10* | higher expression in receptacle |  | Li et al., 2016 |
|  |  | *CiARF18* | high expression in stamens and receptacles |  |  |
| **Fruit** | ***Solanum lycopersicum*** | *SlARF3* | developmental timing changes; formation of epidermal cells and trichomes |  | Zhang et al 2015b; Sessions et al., 1997 |
|  |  | *SlARF4* | sugar metabolism during fruit development; improves fruits resistance to water deficit | SlABI5/ABF/SCL3 | Sagar et al 2013; Chen et al 2021 |
|  |  | *SlARF6/8* | controlling growth and development of vegetative | MiR167a | Liu et al 2014a |
|  |  | *SlARF7* | negative regulator of fruit set; moderates the auxin response; mediates crosstalk between auxin and gibberellin signaling during fruit set and development |  | De Jong et al 2009; De Jong et al 2011 |
|  |  | *SlARF9* | regulate fruit cell division |  | De Jong et al., 2015 |
|  |  | *SlARF10/12* | high expression in fruit species during early development | miR160 | Kumar et al., 2011; Hendelman et al., 2012 |
|  | ***Pyrus pyrifolia*** | *PpARF1* | high expression in fruits and seeds of hardcore stage |  | Diao et al., 2020 |
|  |  | *PpARF1-6* | regulating fruit development |  |  |
|  | ***Citrus sinensis*** | *CiARF1/2/17/18* | regulate pulp development |  | Li et al., 2016 |
|  |  | *CiARF8/12/14* | high expression in fruit |  |  |
|  |  | *CiARF6/7/12/18* | regulation of pericarp development |  |  |
|  |  | *CiARF17* | higher expression on ovary |  |  |
|  | ***Vitis vinifera*** | *VvARF1/5/15/16/18* | regulate fruit development |  | Wan et al., 2014 |
|  |  | *VvARF3/11* | high expression during fruit ripening |  |  |
|  |  | *VvARF5/15* | high expression in early fruit development |  |  |
|  | ***Malus domestica*** | *MdARF2* | regulation of anthocyanin accumulation in fruit |  | Wang et al., 2020 |
|  |  | *MdARF13* |  | MdIAA121 | Wang et al., 2018 |
