# Supplementary material for: Identification and characterization of auxin response factor (ARF) family members involved in fig (Ficus carica L.) fruit development
Source: PeerJ. 2022 Jul 22;10:e13798. doi: 10.7717/peerj.13798 (PMC9310797; doi:10.7717/peerj.13798)
Supplement: Supplemental Information 8 [file peerj-10-13798-s008.docx]

**Supplementary Table S2. The gene names and ID of ARF gene family in common model plants and friut trees**

| **Gene name** | **Gene ID** |
| --- | --- |
| *AtARF10* | At2g28350 |
| *AtARF16* | At4g30080 |
| *AtARF11* | At2g46530 |
| *AtARF7* | At1g30330 |
| *AtARF19* | At1g19220 |
| *OsARF6* | AK070569 |
| *OsARF12* | AK071455 |
| *OsARF 16* | AK103327 |
| *OsARF17* | AK103280 |
| *OsARF 25* | AK121703 |
| *CiARF7* | Cs4g04520.1 |
| *CiARF9* | Cs5g01980.1 |
| *CiARF16* | Cs7g25670.2 |
| *CiARF19* | Cs4g07020.1 |
| *NtARF1* | Nitab4.5_0000798g0140.1 |
| *NtARF2* | Nitab4.5_0002367g0010.1 |
| *NtARF3* | Nitab4.5_0000663g0220.1 |
| *NtARF4* | Nitab4.5_0001763g0030.1 |
| *NtARF5* | Nitab4.5_0009686g0010.1 |
| *NtARF6* | Nitab4.5_0008016g0010.1 |
| *NtARF7* | Nitab4.5_0002261g0030.1 |
| *NtARF8* | Nitab4.5_0008892g0020.1 |
| *NtARF9* | Nitab4.5_0003119g0050.1 |
| *NtARF10* | Nitab4.5_0013336g0010.1 |
| *NtARF11* | Nitab4.5_0000650g0030.1 |
| *NtARF12* | Nitab4.5_0006740g0010.1 |
| *NtARF13* | Nitab4.5_0002104g0010.1 |
| *NtARF15* | Nitab4.5_0001535g0030.1 |
| *NtARF16* | Nitab4.5_0001988g0030.1 |
| *NtARF17* | Nitab4.5_0000899g0130.1 |
| *NtARF18* | Nitab4.5_0000404g0210.1 |
| *NtARF19* | Nitab4.5_0004188g0050.1 |
| *NtARF21* | Nitab4.5_0001843g0120.1 |
| *NtARF22* | Nitab4.5_0000315g0090.1 |
| *NtARF23* | Nitab4.5_0002071g0010.1 |
| *NtARF24* | Nitab4.5_0003016g0030.1 |
| *NtARF25* | Nitab4.5_0007373g0010.1 |
| *NtARF26* | Nitab4.5_0000304g0070.1 |
| *NtARF27* | Nitab4.5_0000235g0020.1 |
| *NtARF28* | Nitab4.5_0001565g0070.1 |
| *NtARF29* | Nitab4.5_0009109g0060.1 |
| *NtARF30* | Nitab4.5_0002524g0040.1 |
| *NtARF32* | Nitab4.5_0003923g0040.1 |
| *NtARF33* | Nitab4.5_0012799g0010.1 |
| *NtARF34* | Nitab4.5_0000754g0240.1 |
| *NtARF35* | Nitab4.5_0005490g0020.1 |
| *NtARF36* | Nitab4.5_0000106g0200.1 |
| *NtARF37* | Nitab4.5_0000602g0120.1 |
| *NtARF38* | Nitab4.5_0009330g0010.1 |
| *NtARF39* | Nitab4.5_0004657g0030.1 |
| *NtARF42* | Nitab4.5_0000051g0180.1 |
| *NtARF43* | Nitab4.5_0004083g0030.1 |
| *NtARF44* | Nitab4.5_0013243g0020.1 |
| *NtARF45* | Nitab4.5_0004657g0020.1 |
| *NtARF46* | Nitab4.5_0000476g0080.1 |
| *CiARF1* | Cs03g01570.1 |
| *CiARF2* | Cs7g19770.1 |
| *CiARF3* | Cs3g05470.1 |
| *CiARF4* | Cs8g16930.1 |
| *CiARF5* | Cs3g25860.1 |
| *CiARF6* | Cs2g09440.2 |
| *CiARF10* | Cs2g15130.1 |
| *CiARF15* | Cs8g16440.1 |
| *CiARF17* | Cs3g18940.1 |
| *CiARF18* | orange1.1t00508.1 |
| *AtARF5* | At1g19850 |
| *AtARF7* | At1g30330 |
| *AtARF19* | At1g19220 |
| *NtARF1* | Nitab4.5_0000798g0140.1 |
| *NtARF2* | Nitab4.5_0002367g0010.1 |
| *NtARF4* | Nitab4.5_0001763g0030.1 |
| *NtARF5* | Nitab4.5_0009686g0010.1 |
| *NtARF6* | Nitab4.5_0008016g0010.1 |
| *NtARF7* | Nitab4.5_0002261g0030.1 |
| *NtARF8* | Nitab4.5_0008892g0020.1 |
| *NtARF9* | Nitab4.5_0003119g0050.1 |
| *NtARF10* | Nitab4.5_0013336g0010.1 |
| *NtARF11* | Nitab4.5_0000650g0030.1 |
| *NtARF12* | Nitab4.5_0006740g0010.1 |
| *NtARF13* | Nitab4.5_0002104g0010.1 |
| *NtARF14* | Nitab4.5_0000999g0120.1 |
| *NtARF15* | Nitab4.5_0001535g0030.1 |
| *NtARF16* | Nitab4.5_0001988g0030.1 |
| *NtARF17* | Nitab4.5_0000899g0130.1 |
| *NtARF18* | Nitab4.5_0000404g0210.1 |
| *NtARF19* | Nitab4.5_0004188g0050.1 |
| *NtARF21* | Nitab4.5_0001843g0120.1 |
| *NtARF22* | Nitab4.5_0000315g0090.1 |
| *NtARF23* | Nitab4.5_0002071g0010.1 |
| *NtARF24* | Nitab4.5_0003016g0030.1 |
| *NtARF25* | Nitab4.5_0007373g0010.1 |
| *NtARF26* | Nitab4.5_0000304g0070.1 |
| *NtARF27* | Nitab4.5_0000235g0020.1 |
| *NtARF28* | Nitab4.5_0001565g0070.1 |
| *NtARF29* | Nitab4.5_0009109g0060.1 |
| *NtARF30* | Nitab4.5_0002524g0040.1 |
| *NtARF31* | Nitab4.5_0000071g0020.1 |
| *NtARF32* | Nitab4.5_0003923g0040.1 |
| *NtARF33* | Nitab4.5_0012799g0010.1 |
| *NtARF35* | Nitab4.5_0005490g0020.1 |
| *NtARF36* | Nitab4.5_0000106g0200.1 |
| *NtARF37* | Nitab4.5_0000602g0120.1 |
| *NtARF38* | Nitab4.5_0009330g0010.1 |
| *NtARF39* | Nitab4.5_0004657g0030.1 |
| *NtARF43* | Nitab4.5_0004083g0030.1 |
| *NtARF44* | Nitab4.5_0013243g0020.1 |
| *NtARF45* | Nitab4.5_0004657g0020.1 |
| *NtARF46* | Nitab4.5_0000476g0080.1 |
| *NtARF1* | Nitab4.5_0000798g0140.1 |
| *NtARF2* | Nitab4.5_0002367g0010.1 |
| *NtARF3* | Nitab4.5_0000663g0220.1 |
| *NtARF4* | Nitab4.5_0001763g0030.1 |
| *NtARF5* | Nitab4.5_0009686g0010.1 |
| *NtARF6* | Nitab4.5_0008016g0010.1 |
| *NtARF7* | Nitab4.5_0002261g0030.1 |
| *NtARF8* | Nitab4.5_0008892g0020.1 |
| *NtARF9* | Nitab4.5_0003119g0050.1 |
| *NtARF10* | Nitab4.5_0013336g0010.1 |
| *NtARF11* | Nitab4.5_0000650g0030.1 |
| *NtARF12* | Nitab4.5_0006740g0010.1 |
| *NtARF13* | Nitab4.5_0002104g0010.1 |
| *NtARF14* | Nitab4.5_0000999g0120.1 |
| *NtARF15* | Nitab4.5_0001535g0030.1 |
| *NtARF16* | Nitab4.5_0001988g0030.1 |
| *NtARF17* | Nitab4.5_0000899g0130.1 |
| *NtARF18* | Nitab4.5_0000404g0210.1 |
| *NtARF19* | Nitab4.5_0004188g0050.1 |
| *NtARF21* | Nitab4.5_0001843g0120.1 |
| *NtARF22* | Nitab4.5_0000315g0090.1 |
| *NtARF23* | Nitab4.5_0002071g0010.1 |
| *NtARF24* | Nitab4.5_0003016g0030.1 |
| *NtARF25* | Nitab4.5_0007373g0010.1 |
| *NtARF26* | Nitab4.5_0000304g0070.1 |
| *NtARF27* | Nitab4.5_0000235g0020.1 |
| *NtARF28* | Nitab4.5_0001565g0070.1 |
| *NtARF29* | Nitab4.5_0009109g0060.1 |
| *NtARF30* | Nitab4.5_0002524g0040.1 |
| *NtARF31* | Nitab4.5_0000071g0020.1 |
| *NtARF32* | Nitab4.5_0003923g0040.1 |
| *NtARF33* | Nitab4.5_0012799g0010.1 |
| *NtARF34* | Nitab4.5_0000754g0240.1 |
| *NtARF35* | Nitab4.5_0005490g0020.1 |
| *NtARF36* | Nitab4.5_0000106g0200.1 |
| *NtARF37* | Nitab4.5_0000602g0120.1 |
| *NtARF39* | Nitab4.5_0004657g0030.1 |
| *NtARF42* | Nitab4.5_0000051g0180.1 |
| *NtARF43* | Nitab4.5_0004083g0030.1 |
| *NtARF44* | Nitab4.5_0013243g0020.1 |
| *NtARF45* | Nitab4.5_0004657g0020.1 |
| *NtARF46* | Nitab4.5_0000476g0080.1 |
| *OsARF19* | AK103312 |
| *AtARF1* | At1g59750 |
| *AtARF2* | A15g62000 |
| *AtARF3* | At2g33860 |
| *AtARF4* | At5g60450 |
| *AtARF5* | At1g19850 |
| *AtARF17* | At1g77850 |
| *AtARF6* | At1g19850 |
| *AtARF8* | At1g19220 |
| *AtARF12* | At1g34310 |
| *PmARF1* | Pm000994 |
| *PmARF2* | Pm003827 |
| *PmARF5* | Pm010467 |
| *PmARF6* | Pm012968 |
| *PmARF7* | Pm014818 |
| *PmARF13* | Pm024588 |
| *PmARF17* | Pm031349 |
| *CiARF3* | Cs3g05470.1 |
| *CiARF5* | Cs3g25860.1 |
| *CiARF6* | Cs2g09440.2 |
| *CiARF7* | Cs4g04520.1 |
| *CiARF9* | Cs5g01980.1 |
| *CiARF10* | Cs2g15130.1 |
| *CiARF18* | orange1.1t00508.1 |
| *PmARF11* | Pm019941 |
| *SlARF3* | DQ340254.1 |
| *SlARF4* | DQ340259.1 |
| *SlARF6* | HM594684.1 |
| *SlARF8* | EF66734F2.1 |
| *SlARF7* | EF121545.1 |
| *SlARF9* | HM037250.1 |
| *SlARF10* | HM143941.1 |
| *SlARF12* | HM565127.1 |
| *PpARF1* | XM_007225091.2 |
| *PpARF1* | XM_007225091.2 |
| *PpARF2* | XM_020563715.1 |
| *PpARF3* | XM_007213730.2 |
| *PpARF4* | XM_020565141.1 |
| *PpARF5* | XM_020559232.1 |
| *PpARF6* | XM_020562547.1 |
| *CiARF1* | Cs03g01570.1 |
| *CiARF2* | Cs7g19770.1 |
| *CiARF17* | Cs3g18940.1 |
| *CiARF18* | orange1.1t00508.1 |
| *CiARF8* | Cs6g16030.2 |
| *CiARF12* | Cs5g32400.1 |
| *CiARF14* | Cs7g02210.1 |
| *CiARF6* | Cs2g09440.2 |
| *CiARF7* | Cs4g04520.1 |
| *CiARF12* | Cs5g32400.1 |
| *CiARF18* | orange1.1t00508.1 |
| *CiARF17* | Cs3g18940.1 |
| *VvARF1* | XM_002268813 |
| *VvARF 5* | XM_002284983 |
| *VvARF 15* | XM_002264036 |
| *VvARF16* | XM_002284507 |
| *VvARF18* | XM_003634334 |
| *VvARF3* | XM_002266642 |
| *VvARF11* | XM_002266567 |
| *VvARF5* | XM_002284983 |
| *VvARF15* | XM_002264036 |
