# Supplementary material for: Identification and characterization of auxin response factor (ARF) family members involved in fig (Ficus carica L.) fruit development
Source: PeerJ. 2022 Jul 22;10:e13798. doi: 10.7717/peerj.13798 (PMC9310797; doi:10.7717/peerj.13798)
Supplement: Supplemental Information 9 [file peerj-10-13798-s009.docx]

**Supplementary Table S3.** **Sequences of 15 predicted motifs of FcARF proteins.**

| Motif | Best possible match | E-value | Width | Mumbers of *FcARF* protein conserved motifs |
| --- | --- | --- | --- | --- |
| 1 | A[KR]D[LV]HGNEW[KR]FRHI[FY]RGQP[RK]RHLL[TQ][TS]GWSVFc[SN][SA]K[RK]LVAGD[SA][VF][ILV]F[LI]R[GA]E[NK] | 3.1E-590 | 18 | 2,6,1,15,8,12,11,9,14,20,13,3,4,7,16,5,10,17 |
| 2 | FCKTLT[AQ]SDT[SN][TN][HG]GGFSV[PL]R[RY][AH]A[ED][KET][FV][FL]PPLD[YM]SQQPPAQEL | 8.8E-428 | 17 | 15,8,19,6,5,1,2,7,20,3,14,18,9,13,16,10,17 |
| 3 | [KP]WR[SCL]L[KQ]V[RG]WDE[PST][STD][AS][IG][EP][RN][PQV][ED]RVSPW[EKL]IEP[VLA] | 1.00E-274 | 19 | 12,11,2,20,9,15,8,19,6,7,18,1,4,3,16,17,13,5,10 |
| 4 | LWHACAG[PG][LM][VA][ST][LV]P[PLR]V[GN][SE][KRL]V[FYV]YFPQGH[SA]E[QH] | 1.40E-252 | 16 | 1,2,15,8,5,19,13,14,20,7,16,9,3,4,10,17 |
| 5 | W[QK][LVI][VL][YF][TV]DDE[NG]D[VM][ML]LVGDDPWQEF[CV][NK]X[VA]R[KR]I[FK]I[LY][ST]P | 1.50E-245 | 16 | 7,2,18,1,5,15,8,4,3,9,19,6,14,13,16,17 |
| 6 | GMRF[KR]MRFE[TG]E[ED][SA][PS][ERV][RIQ][RS][FYW][MST]G[TG] | 7.00E-216 | 20 | 2,9,19,20,12,11,15,6,8,7,5,1,16,13,4,3,10,18,14,17 |
| 7 | NLP[PS][KQ][IL][LI]CQ[VL][HV]NV[TQ][LM][HK]A[DE][PV][ED]TDEVYAQ[MI]T | 6.10E-192 | 14 | 15,8,7,19,18,1,20,6,2,9,14,5,3,4 |
| 8 | A[TA]A[AW]HA[AI][AS][TN][GN][ST]PFT[VI][FY]Y[KN]PR[AT] | 3.20E-169 | 19 | 2,15,6,8,7,12,11,19,4,5,20,18,1,9,14,13,16,10,17,3 |
| 9 | [AS][VL]GR[SA][VLI]D[LI][ST][KR][FL]S[GS]YD[ED]L[LR][ARS]EL[AED][RE][ML]F[GDE][IFL][EG]G[EQS]L | 1.60E-223 | 19 | 18,1,12,6,15,8,2,19,11,7,20,14,9,4,3,5,13,16,17 |
| 10 | L[RL][VL]G[VI]RRA[MN]R[QPK][QP][STD][NV][MLV][PS]SSV[IL]SS[DH][SN]MH[LI]G[VL] | 6.30E-183 | 14 | 2,12,11,18,1,15,8,20,6,7,5,9,4,19,3 |
| 11 | [IV][TI][GS][IV][ES]D[LA]D[PS]VRW[PK][ND]S | 6.00E-92 | 17 | 15,8,7,6,4,3,2,19,12,11,5,20,14,13,10,16,17 |
| 12 | [SA]EF[VI][VI]PL[AD][KR]YM[KEA][AS]VY | 2.60E-87 | 20 | 15,8,5,7,19,6,12,11,2,4,3,20,14,18,1,16,10,9,13,17 |
| 13 | GKQQSX[SP]TR[ST]CTKVHKQG | 6.20E-81 | 16 | 2,12,11,4,3,18,1,20,9,15,8,19,14,5,7,6 |
| 14 | QKYEAVK[PL]KEGNCKLFGYSLIRPVEKFA[RG]Q[MT]NH[MV]SSQAHKFEFTQKSEQA | 9.20E-57 | 3 | 11,12,2 |
| 15 | ISHQAQG[RK]Y[VA]GFDDHPILHGH[WR]VEHPHGN[RW]F[IM]PPPSSP[CH]LE | 1.80E-44 | 3 | 12,11,2 |
