# Supplementary material for: Identification and characterization of auxin response factor (ARF) family members involved in fig (Ficus carica L.) fruit development
Source: PeerJ. 2022 Jul 22;10:e13798. doi: 10.7717/peerj.13798 (PMC9310797; doi:10.7717/peerj.13798)
Supplement: Supplemental Information 10 [file peerj-10-13798-s010.docx]

**Supplementary Table S4. Ka/Ks analysis for the ARF duplicated genes**

| Gene name | Gene name | Ka | Ks | Ka/Ks |
| --- | --- | --- | --- | --- |
| *FcARF16* | *FcARF5* | 1.00806 | 0.972866 | 1.03618 |
| *FcARF17* | *FcARF13* | 1.01515 | 0.95445 | 1.0636 |
| *FcARF17* | *FcARF3* | 1.00523 | 0.984027 | 1.02155 |
| *FcARF17* | *FcARF4* | 0.947653 | 1.17108 | 0.809212 |
| *FcARF17* | *FcARF18* | 1.00613 | 0.981478 | 1.02512 |
| *FcARF17* | *FcARF1* | 0.975594 | 1.07994 | 0.903374 |
| *FcARF17* | *FcARF11* | 1.02042 | 0.935034 | 1.09132 |
| *FcARF17* | *FcARF2* | 0.999854 | 1.00044 | 0.999411 |
| *FcARF17* | *FcARF20* | 1.00525 | 0.983843 | 1.02176 |
| *FcARF17* | *FcARF14* | 0.987592 | 1.0392 | 0.950336 |
| *FcARF17* | *FcARF5* | 0.994761 | 1.01631 | 0.978797 |
| *FcARF13* | *FcARF10* | 0.992916 | 1.02315 | 0.970452 |
| *FcARF13* | *FcARF3* | 0.958801 | 1.14201 | 0.839577 |
| *FcARF13* | *FcARF4* | 0.966794 | 1.12793 | 0.857141 |
| *FcARF13* | *FcARF1* | 0.995676 | 1.01536 | 0.98061 |
| *FcARF13* | *FcARF20* | 1.00836 | 0.972352 | 1.03704 |
| *FcARF13* | *FcARF14* | 1.01293 | 0.956099 | 1.05944 |
| *FcARF13* | *FcARF5* | 0.991645 | 1.02924 | 0.963474 |
| *FcARF10* | *FcARF3* | 1.00124 | 0.995717 | 1.00555 |
| *FcARF10* | *FcARF4* | 0.979507 | 1.064 | 0.920588 |
| *FcARF10* | *FcARF18* | 1.03265 | 0.893887 | 1.15523 |
| *FcARF10* | *FcARF1* | 0.986053 | 1.04878 | 0.940192 |
| *FcARF10* | *FcARF11* | 0.98542 | 1.051 | 0.937606 |
| *FcARF10* | *FcARF2* | 0.999442 | 1.00187 | 0.997572 |
| *FcARF10* | *FcARF20* | 0.993206 | 1.02248 | 0.971365 |
| *FcARF10* | *FcARF14* | 0.966597 | 1.11286 | 0.868573 |
| *FcARF10* | *FcARF5* | 1.0056 | 0.981319 | 1.02475 |
| *FcARF3* | *FcARF4* | 1.02755 | 0.90739 | 1.13242 |
| *FcARF3* | *FcARF1* | 1.02209 | 0.920441 | 1.11043 |
| *FcARF3* | *FcARF20* | 0.936343 | 1.21913 | 0.768041 |
| *FcARF3* | *FcARF14* | 1.00792 | 0.972271 | 1.03667 |
| *FcARF3* | *FcARF5* | 1.0112 | 0.959369 | 1.05403 |
| *FcARF4* | *FcARF1* | 0.98324 | 1.05459 | 0.932348 |
| *FcARF4* | *FcARF20* | 0.975555 | 1.09429 | 0.891499 |
| *FcARF4* | *FcARF14* | 1.016 | 0.941485 | 1.07915 |
| *FcARF4* | *FcARF5* | 0.994969 | 1.01678 | 0.978548 |
