# Supplementary material for: Identification and characterization of auxin response factor (ARF) family members involved in fig (Ficus carica L.) fruit development
Source: PeerJ. 2022 Jul 22;10:e13798. doi: 10.7717/peerj.13798 (PMC9310797; doi:10.7717/peerj.13798)
Supplement: Supplemental Information 11 [file peerj-10-13798-s011.docx]

**Supplementary Table S5. Primers sequence used in amplification, qPCR**

| **No.** | **Named** | **Primers Sequence** | |
| --- | --- | --- | --- |
|  |  | **Forward (5’-3’)** | **Reverse (5’-3’)** |
| **1** | **qPCR -** FcARF1 | CTCGGCTACGAATCATGCCT | AAGGCATTTGCTGCTCTTGC |
| **2** | **qPCR -** FcARF2 | CACTTTGCTGCCTGAACACC | CAGTGGGGGAAGGCATTCAT |
| **3** | **qPCR -** FcARF3 | AGGTGCAGTGGCGTAGTAAC | GTGGGAGAGAAACCGAAGGG |
| **4** | **qPCR -** FcARF4 | AAAGAAGGTGCAGTGGCGTA | GGGTGGGAGAGAAACCGAAG |
| **5** | **qPCR -** FcARF5 | TCATAGCGAGCAAGTAGCGG | TCGGCTTCAGTCCAAAGTCC |
| **6** | **qPCR -** FcARF7 | TCATGGAGGTTTCTCGGTGC | CAGAGTCACCGGCAAAAAGC |
| **7** | **qPCR -** FcARF8 | TGCACATCGGTCTTCTTGCT | CAAACAGCATCCTGAACCGC |
| **8** | **qPCR -** FcARF9 | ATAGCATGCACTTGGGGCTT | CGAACAGGATCTAGGTCGCC |
| **9** | **qPCR -** FcARF10 | AGGATGTTCACGGCGAAAGT | TGGATGGTTCCAACGGGAAG |
| **10** | **qPCR -** FcARF11 | CGACGATACCACGTCCAGAG | CGGGCCTTGATAACCCACTT |
| **11** | **qPCR -** FcARF12 | CGACGATACCACGTCCAGAG | CGGGCCTTGATAACCCACTT |
| **12** | **qPCR -** FcARF13 | ATTGTGCTGTGCCTTACGGA | CCCGTTGGAAGCAAGTGTTG |
| **13** | **qPCR -** FcARF14 | CGTCCACGTAACAAGTGGGA | TACGGTCCCTTCGGCTTCTA |
| **14** | **qPCR -** FcARF15 | TTGAAGGCCAACTGGAGGAC | TCTTTGCCCATTTGCTGCAC |
| **15** | **qPCR -** FcARF16 | TTCGCCAAGATGAAGCTCGT | GGATCCGCCGAGTAATCCAG |
| **16** | **qPCR -** FcARF17 | CACCCAATCGGACTCCAACA | TCCTCTCGTTGACGAACACG |
| **17** | **qPCR -** FcARF18 | ATGGCAGGTTGTCTACACCG | CCGGTTTGGCGTCTTGATTG |
| **18** | **qPCR -** FcARF19 | GCTGGGAGACGACTTTGGAA | CAGCACTGAGGTTGTTGTGC |
| **19** | **qPCR -** FcARF20 | CTTCGTGTAGGGGTCAGACG | AGCGCTTATCCTTGGCTTGT |
| **20** | **qPCR -** FcARF21 | CTCGCCAACTTCCACAGCTA | GGTGGACTTGAGTGACCCTG |
| **21** | **Actin** | GAACCACCAGACAGGACGATG | CTACCACTGCTGAACGGGAAA |
| **22** | **18S rRNA** | TCAAGGAAAGACAACGAGACGAT | GGATTCTGCAATTCACACCAAG |
